# Effect of Prehabilitation on the 6-Minute Walk Test and Length of Hospital Stay in Frail Older People: A Meta-Analysis of Randomized Controlled Trials

**DOI:** 10.3390/ijerph22091381

**Published:** 2025-09-03

**Authors:** María López-González, Celia Álvarez-Bueno, Beatriz Rodríguez-Martín, Patricia Lorenzo-García, Marta Carolina Ruiz-Grao, Susana Priego-Jiménez

**Affiliations:** 1 Age-ABC Research Group, Health and Social Research Center, University of Castilla-La Mancha, 13001 Ciudad Real, Spain; maria.lopezgonzalez@uclm.es (M.L.-G.); beatriz.rmartin@uclm.es (B.R.-M.); patricia.garcia@uclm.es (P.L.-G.); marta.ruiz@uclm.es (M.C.R.-G.); susiprie@hotmail.com (S.P.-J.); 2 Faculty of Health Sciences, Universidad Autónoma de Chile, Talca 7500912, Chile; 3 Network for Research on Chronicity, Primary Care, and Health Promotion (RICAPPS), Faculty of Health Sciences, University of Castilla-La Mancha, 45600 Toledo, Spain; 4 FISANA Physiotherapy Center, Ronda de Belén Street 1., 45450 Toledo, Spain; 5 Nursing Faculty, University of Castilla-La Mancha, 02071 Albacete, Spain; 6 Virgen de la Luz Hospital, C/Hermandad de Donantes de Sangre 1, 16002 Cuenca, Spain

**Keywords:** frail elderly, exercise capacity, aerobic capacity, preoperative prehabilitation, length of hospital stay, postoperative complications, meta-analysis

## Abstract

Frailty reduces resilience to surgical stress, increasing vulnerability to adverse outcomes. While recovery efforts traditionally focus on the postoperative phase, the preoperative period offers better opportunities for lifestyle interventions. Prehabilitation aims to increase functional reserve and surgical tolerance, especially in frail older adults. This systematic review and meta-analysis of randomized controlled trials (RCTs) evaluated the effectiveness of multimodal prehabilitation on aerobic capacity—measured by the 6 min walk test (6MWT)—and the length of hospital stay (LOS). A literature search was conducted up to August 2025. Eligible RCTs reported the effects of prehabilitation on functional capacity and LOS. A pairwise meta-analysis was used to calculate pooled mean differences (p-MDs) with 95% confidence intervals (CIs). The risk of bias was assessed via the Cochrane RoB tool, and evidence quality was assessed via the GRADE system. Five studies involving 400 participants were included. The p-MD for the 6MWT showed no significant improvement at any time point: (T1–T2) 9.71 (CI: −38.92; 58.36), (T2–T3) −3.27 (CI: −71.21; 64.65), and (T1–T3) 15.01 (CI: −22.05; 52.07). The LOS was also not significantly reduced (p-MD: −0.464, CI: −0.960; 0.031). Prehabilitation did not significantly improve aerobic capacity or reduce hospital stay. Future research should explore long-term benefits and adherence.

## 1. Introduction

In general, frailty is a multidimensional clinical syndrome characterized by a reduced reserve and function due to the dysregulation of physiological and molecular pathways, which can lead to vulnerability in multiple systems and organs [1]. Various general assessment tools have been used to measure the term “frailty” due to the lack of uniform consensus on its definition or on the best way to measure it [2]. Although aging does not necessarily imply frailty, older adults are more likely to be frail, increasing their susceptibility to negative health outcomes [3]. Frail patients are generally more susceptible to stressors, and even mild illnesses in this age group can cause a significant deterioration in their well-being [4].

When undergoing surgery, frail older adults have limited physiological reserves to withstand the impending surgical stress response, making them particularly vulnerable to negative effects, including postoperative complications and prolonged hospital stays [5,6]. Even in the absence of complications, the postoperative period is associated with a 20–40% reduction in physiological and functional capacity, which, particularly in older adults with comorbidities, may not return to presurgical levels for several months, if at all [7]. Therefore, surgery presents a significant challenge for frail patients and is a better predictor of adverse surgical outcomes than age is [8].

Traditionally, efforts to improve health recovery have focused on the postoperative period (rehabilitation). However, this may not be the most suitable time to initiate lifestyle changes, as patients are often fatigued, are concerned about affecting the healing process, or are anxious while awaiting further treatment for the underlying disease [9]. Therefore, the preoperative period may represent a more appropriate time than the postoperative period to implement an intervention [10]. Prehabilitation, defined as a multidisciplinary preoperative intervention to prevent or minimize functional decline after surgery and improve postoperative outcomes, has been implemented as a strategy [11]. Preoperative multimodal prehabilitation, which includes moderate-intensity aerobic and resistance exercise, dietary counseling, and anxiety reduction strategies, has been shown to improve preoperative functional capacity and accelerate postoperative recovery [12].

Emerging evidence indicates that a multimodal prehabilitation program improves the preoperative functional reserve and subsequently helps patients better tolerate the side effects of surgery [13]. As a low preoperative physical performance increases the risk of mortality [14] and the number of postoperative complications [15] and prolongs functional recovery [16], prehabilitation could reduce medical costs and increase hospital bed turnover by shortening the length of stay (LOS) [17]. However, conflicting results exist regarding the effects of prehabilitation on postoperative mortality and the incidence of complications in older surgical patients [18]. The evidence on specific interventions to improve outcomes in frail older adults or frail surgical patients is limited, with some studies showing no significant changes with prehabilitation in these groups [19] or failing to demonstrate an improvement in preoperative functional capacity in patients with colorectal cancer [20].

Identifying strategies to improve physiological reserve and function before surgery, as well as maintaining it afterwards, could benefit the frail and vulnerable population. Therefore, the aim of this systematic review and meta-analysis of randomized controlled trials (RCTs) was to determine the effectiveness of prehabilitation on aerobic capacity and the LOS in frail older adults undergoing surgery.

## 2. Materials and Methods

This systematic review and meta-analysis were conducted in accordance with the Cochrane Collaboration Handbook [21] and were reported in accordance with the preferred reporting elements for systematic review (PRISMA) [22] (Table A1). In addition, the study protocol was preregistered in PROSPERO (registration number ID: CRD42025642272).

### 2.1. Search Strategy and Selection Criteria

Two reviewers (M.L.-G. and C.A.-B.) independently searched the PubMed, Web of Science (WoS), Cochrane Central Register of Controlled Trials, and Cochrane Database of Systematic Reviews databases from their inception to August 2025.

This study aimed to identify RCTs to determine the effectiveness of prehabilitation on aerobic capacity measured through the 6-MWT and LOS among frail surgical patients. The search strategy included the following free terms: (“home-based” OR “preoperative prehabilitation” OR “preoperative exercise” OR “preoperative therapeutic”) AND (“comorbidities” OR “postoperative complications” OR mortality) AND (trial). The full search strategy for all the databases is available in the (Table A2). No language limitations were applied. In addition, reference lists of studies and references included in previous systematic reviews and meta-analyses were examined for potentially relevant studies.

The inclusion criteria for the studies were as follows: (1) the type of study was an RCT; (2) the population included people with frailty; (3) the type of intervention included prehabilitation interventions aimed at improving the preoperative aerobic capacity of patients and recovery after surgery; (4) the type of comparison included a control group; and (5) the outcome included aerobic capacity measured through the 6-MWT and LOS.

### 2.2. Study Identification and Data Extraction

Two reviewers (M.L.-G. and C.A.-B.) independently extracted and summarized the following data from each included study in an ad hoc table: (1) study characteristics (author, year of publication, and country); (2) sample characteristics (sample size, mean age, and type of surgery); (3) intervention characteristics (intervention duration and intervention classification); and (4) outcome measurements: 6-MWT and LOS. Disagreements with the selection process and data extraction were resolved by consensus or by discussion with a third reviewer (S.P.-J.).

### 2.3. Risk of Bias

Risk of bias assessment (RoB) was performed by two independent investigators (M.L.-G. and C.A.-B.) via the Cochrane Collaboration’s tool for assessing RoB (RoB2) [23]. Disagreements were resolved by consensus or discussion with a third reviewer (S.P.-J.). This tool assesses the RoB based on five domains. The first domain includes a randomization process; the second, third, fourth and fifth domains include deviations from intended interventions, missing outcome data, outcome measurement, and selection of reported results. The overall RoB was considered ‘low risk’ when the study scored ‘low risk’ in all domains, ‘some concerns’ if at least one of the domains scored ‘some concerns’, and ‘high risk’ if at least one of the domains scored ‘high risk’ or several domains scored ‘some concerns’.

### 2.4. Data Analysis

The mean difference (MD) and 95% confidence intervals (95% CI) for the effect of prehabilitation on the 6-MWT and LOS were calculated via Cohen’s d-index. A pooled MD (p-MD) was estimated via a random-effects model based on Der Simonian and Laird’s method [24] in meters for the 6-MWT and in days for the LOS. Heterogeneity between studies was assessed via the I2 statistic [25], whose values were considered as follows: not important (0–30%), moderate (30–50%), substantial (50–75%), and considerable (75–100%). In addition, the corresponding *p* value was also considered [21].

Two subgroup analyses were developed on the basis of (i) the type of prehabilitation performed, distinguishing between unimodal prehabilitation (considering prehabilitation as a single intervention), bimodal prehabilitation (if it includes two interventions), or trimodal prehabilitation (if it includes three interventions), and (ii) the period when the prehabilitation program was performed: the start of prehabilitation (T1), the period prior to surgery (T2), and post-surgery (T3) [26]. The grouping of the time points T1 (start of prehabilitation—baseline level), T2 (period prior to surgery), and T3 (post-surgery) was defined on the basis of key clinical and temporal phases in the prehabilitation and surgical recovery process. T1 represents the initiation of the prehabilitation intervention, allowing for the assessment of its baseline level; T2 corresponds to the period immediately before surgery, crucial for measuring changes and preparations just prior to the procedure; and T3 covers the postoperative phase, where recovery and the short- to mid-term effects of the intervention are evaluated.

To define the effect of individual studies on the overall MD, a sensitivity analysis was performed. Finally, the effect of small studies was assessed graphically via a funnel plot; in addition, an Egger’s test was performed. All analyses were conducted with Stata/IC software, version 16.1, for Windows.

### 2.5. Grading of Recommendations Assessment, Development, and Evaluation (GRADE)

The Grading of Recommendations Assessment, Development, and Evaluation (GRADE) framework was used to evaluate the certainty of evidence for each outcome of this meta-analysis on the different outcomes and provide recommendations [27]. It can be rated as having a high, moderate, low, or very low evidence value, depending on the study design, risk of bias, inconsistency, indirect evidence, imprecision, and publication bias.

## 3. Results

### 3.1. Study Identification

**Figure 1:**
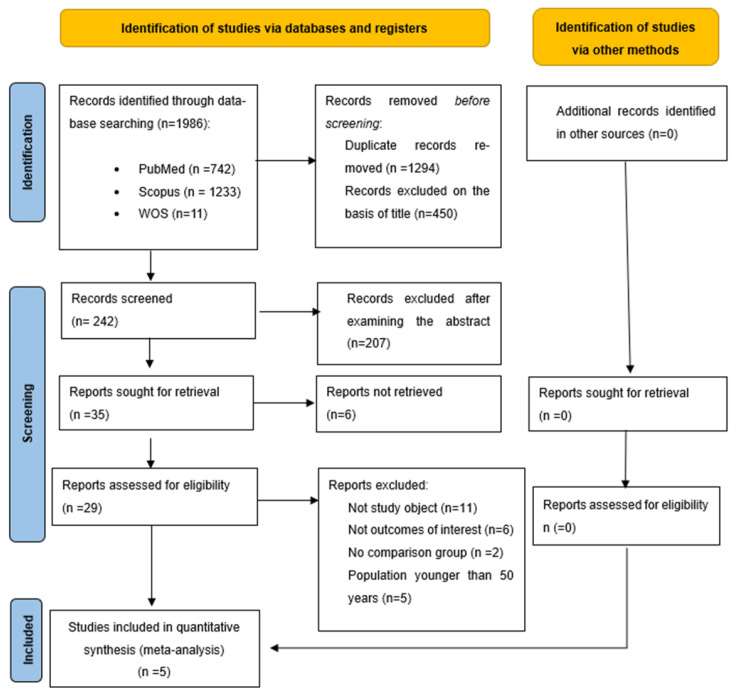
PRISMA 2020 flow diagram for new systematic reviews that include searches of databases, registers, and other sources.

Following the search strategy, 1987 records were found. After removing duplicates and reviewing the title and abstract, 30 relevant articles were selected for full-text reading. Finally, only 5 RCTs met the inclusion criteria (Figure 1).

### 3.2. Characteristics of the Included Studies

Five studies [26,28,29,30,31] assessing the postoperative effectiveness of prehabilitation via the 6-MWT and the reduction in LOS were included in the review. The studies included participants from two countries, the Netherlands [26,28,30] and Canada [29,31]. The studies included participants with ages ranging from 73.6 (SD: 6.1) to 78 (SD:18.9) and 73 (SD:6) to 82 (SD:17.01) in the intervention and control groups, respectively, with women representing 54.34%. (Table 1). All studies included participants who were older adults with frailty, defined via the Groningen Frailty Indicator (GFI), Clinical Frailty Scale (CFS), Fried Frailty Index (FFI), and Frailty Index Identification of Seniors at Risk (ISAR) (Table A3). The duration of the interventions ranged from 2.5 to 6 weeks.

The studies included the following surgical interventions: colon surgery [28,29], intra-abdominal or thoracic cancer [31], and total hip arthroplasty [26,30]. With respect to prehabilitation interventions, all studies included physical exercise [26,28], two included nutrition [29,31], and one study included the psychological component [29] within the three dimensions that prehabilitation addresses. Table 2 provides a complete description of the intervention characteristics.

### 3.3. Risk of Bias

After the RoB was assessed via the Cochrane Collaboration risk of bias (RoB2) tool [23], two studies were evaluated as having a low RoB [29,31], and three studies had some concerns regarding bias [26,28,30].

**Figure 2:**
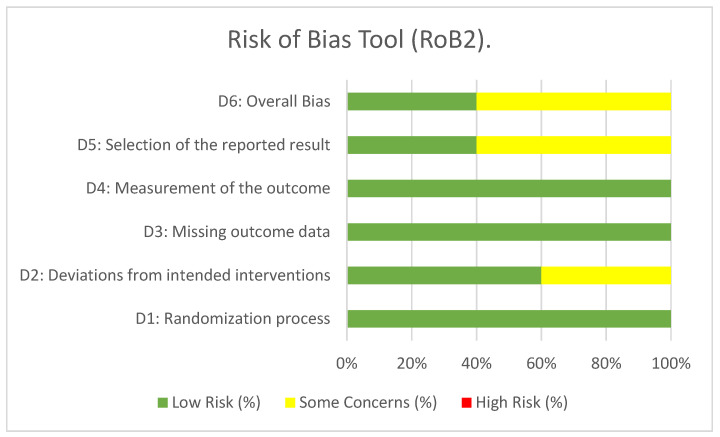
Percentage risk of bias of included studies assessed with the risk of bias tool (RoB2).

**Figure A1:**
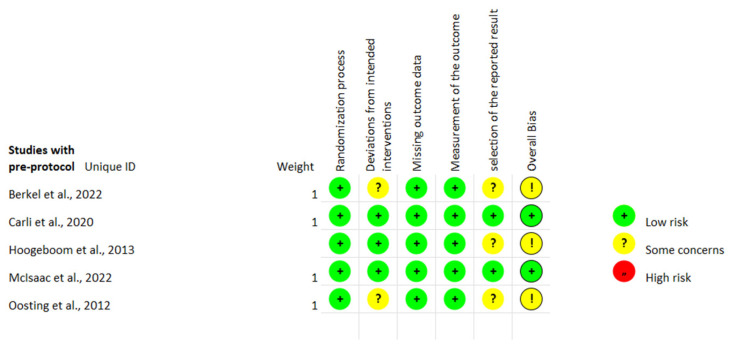
Risk of bias of studies included as assessed with the RoB2 tool [26,28,29,30,31].

In the randomization process, 100% of the studies were classified as having a low risk of bias; deviations from the intended interventions were classified as having a low risk of bias in 60% of the studies; loss of data on outcomes was classified as having a low risk of bias in 100% of the studies; outcome measurement was classified as having a low risk of bias in 100% of the studies; and selection of reported outcomes was classified as having a low risk of bias in 40% of the studies (Figure 2) (Figure A1).

### 3.4. Grading of the Quality of Evidence

Additionally, the GRADE results are available in Table 3 and Table 4, and Table A4 and Table A5. For the 6-MWT, the GRADE assessment was rated as important for T1 and T3 and critical for T2; for the LOS, the GRADE assessment was rated as important.

### 3.5. Data Synthesis

#### Meta-Analysis

**Figure 3:**
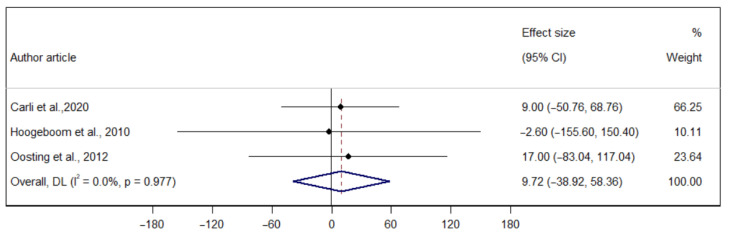
Forest plot of the direct effect of prehabilitation on the 6-MWT (T1–T2) in older adults [26,29,30].

**Figure 4:**
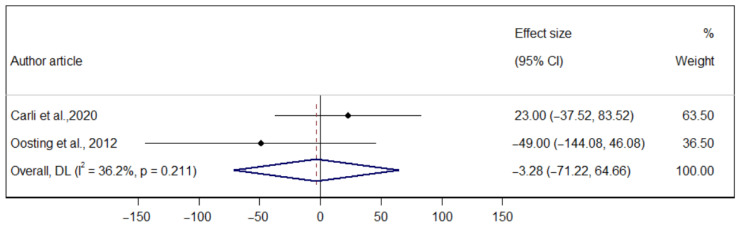
Forest plot of the direct effect of prehabilitation on the 6-MWT (T2–T3) in older adults [26,29].

**Figure 5:**
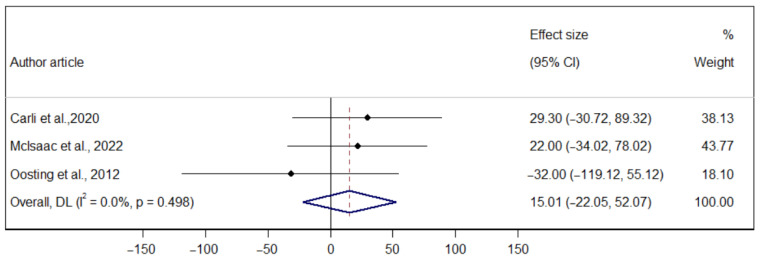
Forest plot of the direct effect of prehabilitation on the 6-MWT (T1–T3) in older adults [26,29,31].

The p-MD of prehabilitation on aerobic capacity measured through the 6-MWT (T1–T2) was 9.71 m (95% CI −38.92; 58.36), indicating no significant heterogeneity (I^2^ = 0.00%). The p-MD of prehabilitation on the 6-MWT (T2–T3) was −3.27 m (95%: IC −71.21; 64.65), indicating considerable heterogeneity (I^2^ = 36.2%). Finally, the p-MD of prehabilitation on the 6-MWT (T1–T3) was 15.01 m (95% CI: −22.05; 52.07), indicating considerable heterogeneity (I^2^ = 0.00%) (Figure 3, Figure 4 and Figure 5).

**Figure 6:**
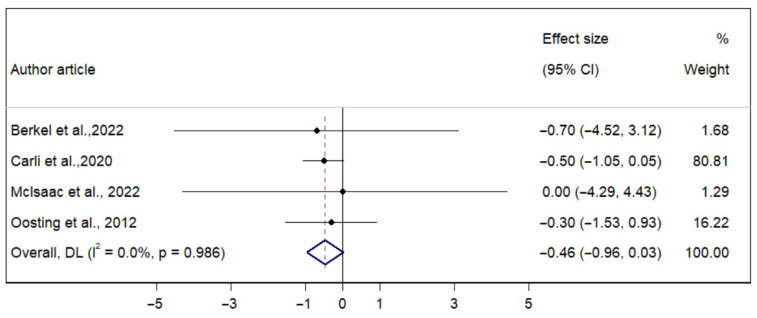
Forest plot of the direct effect of prehabilitation on the LOS in older adults [26,28,29,31].

On the other hand, the p-MD of prehabilitation for LOS was −0.464 days (95% CI: −0.960; 0.031), with no significant heterogeneity (I^2^ = 0.00%) (Figure 6).

### 3.6. Sensitivity Analysis

The sensitivity analysis could only be performed on the aerobic capacity measured through the 6-MWT results at the time points T1–T2 and T1–T3 and for the LOS. After each study was individually removed from the pooled MD, none of the studies substantially altered the pooled MD estimate for the 6-MWT (Table A6) or for the LOS (Table A7).

However, a sensitivity analysis for the 6-MWT at T2–T3 was not possible because of the limited number of reported data.

### 3.7. Publication Bias

**Figure A2:**
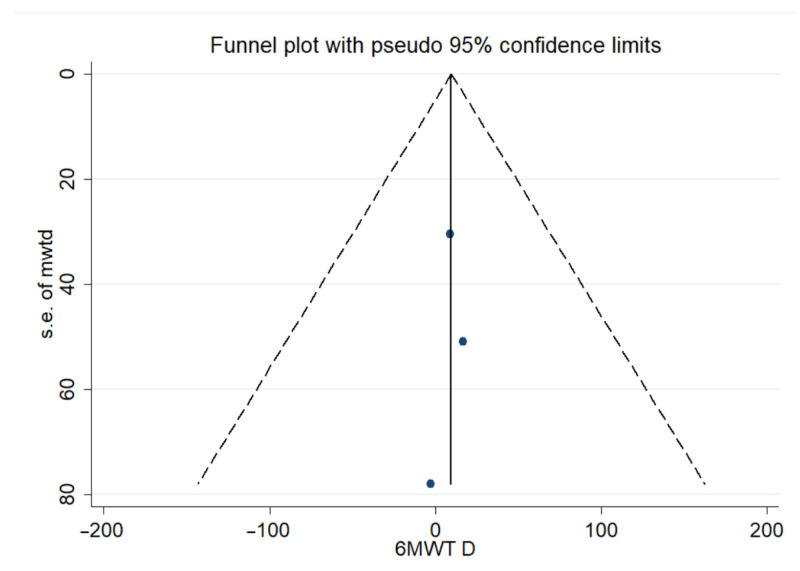
Funnel plot for comparison-specific pooled mean differences on the 6 min walk test. (T1–T2).

**Figure A3:**
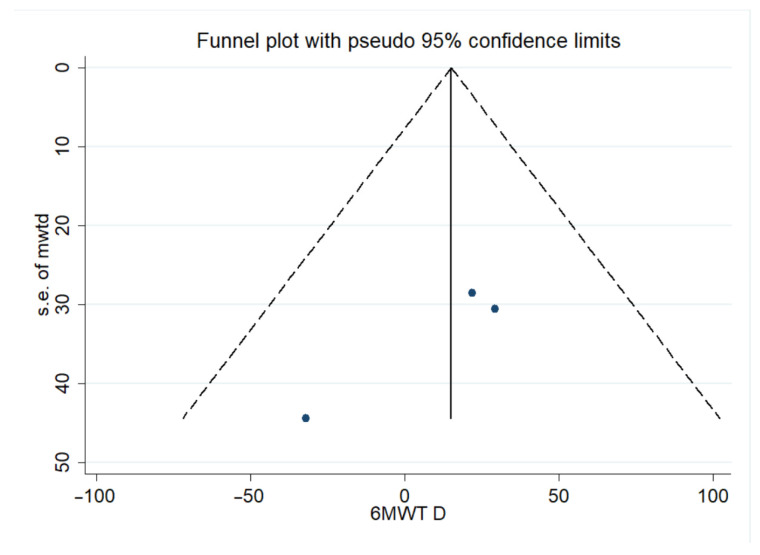
Funnel plot for comparison-specific pooled mean differences on the 6 min walk test. (T1–T3).

**Figure A4:**
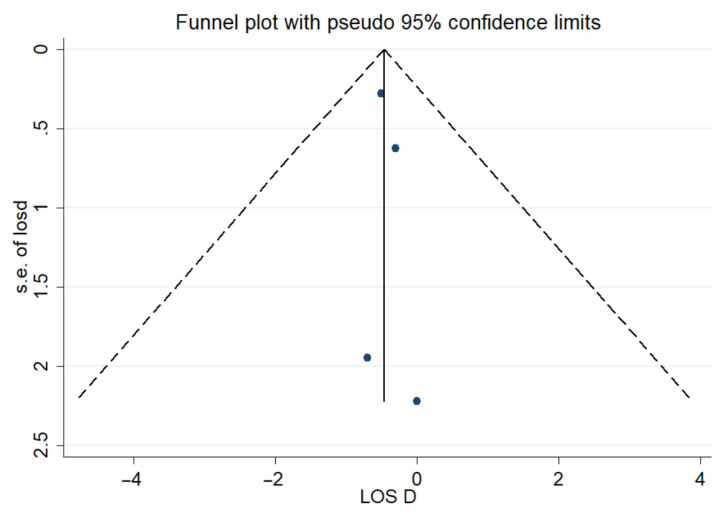
Funnel plot for comparison-specific pooled mean differences on length of hospital stay (LOS).

The Egger’s test for the 6-MWT revealed no publication bias at the time points T1–T2 (*p* = 0.811) and T1–T3 (*p* = 0.198) (Figure A2 and Figure A3). The Egger’s test also revealed no publication bias for the LOS results (*p* = 0.534) (Figure A4).

However, for the 6-MWT (T2–T3), this was not possible because of the limited number of reported data.

## 4. Discussion

This systematic review and meta-analysis aimed to determine the effectiveness of prehabilitation on the aerobic capacity and hospital LOS in older frail adults undergoing surgical intervention. This meta-analysis, which included five RCTs with data from 400 participants, suggested that prehabilitation could be a clinically relevant therapeutic strategy to improve exercise capacity both before and after surgery, as well as to reduce the LOS, although the results did not reach statistical significance. On the other hand, when the pre-surgery period was compared with the postoperative period, no significant effect was observed according to the results of the 6-MWT.

To our knowledge, this is the first systematic review and meta-analysis that evaluated the impact of prehabilitation interventions on pre-surgery and post-surgery exercise capacity, as measured by the 6-MWT test and LOS, in frail older adults undergoing various surgical interventions. It has been shown that preoperative frailty is associated with a greater risk of postoperative complications [32]. The analysis of the available data suggests that prehabilitation is beneficial for improving exercise capacity before surgery. However, it does not appear to have a significant impact when comparing the period prior to surgery with the postoperative period. This could be due to several factors, among which the patients included in this analysis primarily had oncological and orthopedic diseases, which negatively affected their exercise [33].

Therefore, when evaluating outcomes, maintaining patients’ functional capacity could represent a clinically relevant improvement. Consequently, the lack of a significant change in our postsurgical outcomes may be explained by this maintenance approach, rather than a substantial improvement objective encompassing prehabilitation, since participants with total hip and knee prostheses may have been influenced by orthopedic factors, limiting the functionality of our results. Additionally, oncology patients often experience loss of appetite, which can also reduce muscle mass, strength, and functional capacity [34].

Second, the intervention protocol was not clearly defined in the included studies, indicating a lack of a comprehensive approach to prehabilitation interventions. The detailed components of prehabilitation are still in the exploratory phase, without uniform standards, and there is notable heterogeneity in terms of interventions, duration, and intensity, which vary considerably [35]. The short intervention periods in prehabilitation programs may limit their effectiveness, as a minimum number of weeks is required to observe significant improvements in physical and nutritional status. Otherwise, it is difficult to achieve relevant changes within such a short period [36]. Significant increases in functional capacity and muscle strength have been observed in older patients after 12 weeks of prehabilitation, suggesting that longer interventions are needed to demonstrate the benefits of prehabilitation in long-term outcome indicators [37].

Most patients aim to recover their presurgical condition and quickly reinsert themselves into social life to avoid compromising their quality of life [38]. Therefore, prehabilitation should be considered a comprehensive and promising tool for optimizing health before surgery and reducing the LOS [39]. Future research should focus on designing strategies that promote patient adherence to treatment, optimize interventions, and assess the associated costs. This will allow for proper investment and make such strategies a key tool for public health policies directed at patients undergoing different types of surgery.

This study has several limitations that should be considered. First, the results should be interpreted with caution because of the relatively small number of studies included in the systematic review. This limitation may affect both the comprehensiveness of the analysis and the generalizability of the findings. The primary reason for this restriction was the limited available evidence on prehabilitation programs specifically targeting older adults with clinically defined frailty. Second, there was notable heterogeneity among the included studies regarding the type, duration, and intensity of the prehabilitation programs. Interventions ranged from unimodal to trimodal approaches, lasting between 2.5 and 6 weeks, and involved different surgical populations, such as orthopedic and oncologic patients. This lack of standardization complicates the combined interpretation of the results and may obscure the specific benefits of certain types of interventions. Third, the variability of surgical procedures and the significant heterogeneity in the duration and intensity of prehabilitation interventions prevent generalizations about the optimal dosing of these interventions. Fourth, standard indicators such as the 6MWT and LOS were reported in this review, but other patient-centered outcomes, such as quality of life or postoperative complications, were not evaluated due to the lack of consistent data in the included studies; therefore, future research is needed to incorporate these more patient-centered outcomes. Fifth, patient adherence to prehabilitation programs was not thoroughly explored because of the lack of detailed information in the included studies. This absence of data on compliance and adherence monitoring makes it difficult to assess the impact of patient engagement on outcomes. Sixth, it was not possible to determine which type of intervention (unimodal, bimodal, or multimodal) was most effective, as there were insufficient data to conduct subgroup or network analyses. Finally, patient adherence is generally low, making it difficult to generalize findings, as each frail older adult presents a wide range of individual complications.

On the basis of our findings, future research should focus on standardizing prehabilitation protocols, extending the duration of interventions, and broadening outcome measures to include more patient-centered end points. Improving adherence monitoring and enhancing the methodological quality of primary clinical trials are also essential to strengthen the existing evidence base and facilitate the effective integration of prehabilitation into surgical care. Furthermore, future research should incorporate subgroup analyses on the basis of etiology to identify which types of patients may benefit most from specific and personalized interventions.

## 5. Conclusions

The results of this systematic review suggest that prehabilitation had no effect on presurgical exercise capacity, although no effects were observed on the postsurgical exercise capacity or LOS. Relevant studies in the field of frailty remain limited, making it difficult to reach definitive conclusions. Therefore, although prehabilitation may be a valuable tool for improving exercise capacity in older adults undergoing surgical intervention, claims regarding its effectiveness should be considered with caution.

## Figures and Tables

**Table 1:** Characteristics of the population described in the included studies.

		Intervention Characteristics						Outcome		
First Author, Year	Country	N (%Woman)	Age (Years, SD)	Length (Weeks)	Type of Surgery	Fragility	Baseline LOS	Baseline-T1 (6MWT)	T2-(6-MWT)	T3-(6-MWT)
Berkel et al., 2022 [28]	Netherlands	IG: 28 (43%) CG: 29 (52%)	IG: 73.6 (6.1) CG: 73 (6)	3	Colon surgery	GFI	IG: 8.4 (7.4) CG: 9.1 (7)	NA	NA	NA
Carli et al., 2020 [29]	Canada	IG: 55 (47.3%) CG: 55 (58.2%)	IG: 78 (18.9) CG: 82 (17.01)	4	Colon surgery	FFI	IG: 4.75 (1.45) CG: 5.25 (1.47)	IG: 325.3 (144.3) CG: 304 (107.3)	IG: 346.1 (117.8) CG: 315.8 (107.5)	IG: 336.4 (121.8) CG: 286.1 (105.1)
Hoogeboom et al., 2010 [30]	Netherlands	IG: 10 (30%) CG: 11 (40%)	IG: 77 (3) CG: 75 (5)	6	Total hip replacement	CFS	NA	IG: 359.7 (117.4) CG: 336.8 (92.1)	IG:363 (126.63) CG: 342.7 (133.7)	NA
McIsaac et al., 2022 [31]	Canada	IG: 94 (60.6%) CG: 88 (52.3%)	IG: 74 (7) CG: 74 (6)	4	Intra-abdominal or thoracic cancer	CFS	IG: 6 (12.37) CG: 6 (16.75)	IG: 306 (130) CG: 323 (106)	NA	NA
Oosting et al., 2012 [26]	Netherlands	IG: 15 (93%) CG: 15 (67%)	IG: 76.9 (6.3) CG: 75 (6.3)	IG: 37 (9) * CG: 32 (6) *	Total hip arthroplasty	ISAR	IG: 5.1 (1) CG: 5.4 (2.1)	IG: 272 (74) CG: 296 (99)	IG: 288 (88) CG: 296 (113)	IG: 282 (84) CG: 339 (69)

Six min walk test (6-MWT); length of hospital stay (LOS); and * expressed in days. Groningen Frailty Indicator (GFI); Clinical Frailty Scale (CFS); Fried Frailty Index (FFI); and Frailty Index Identification of Seniors at Risk (ISAR). NA = not applicable; T1: start of prehabilitation; T2: period prior to surgery; and T3: post-surgery.

**Table 2:** Characteristics of the interventions described in the included studies.

Study	Groups by Intervention	Intervention	Time (min)/Rep	Intensity	Length (Weeks)	Frequency (x/wk)
**Berkel et al., 2022** [28]	Prehabilitation CON	Exercise (high-intensity aerobics, aerobic fitness, and resistance training) Usual care	40 min aerobic-ex + 20 min strength training	NA	3	3
**Carli et al., 2020** [29]	Prehabilitation CON	Exercise + nutrition (protein intake of 1.5 g/kg of body weight) + psychological Usual care—an identical multimodal program after postoperative hospital discharge	30 min aerobic-ex + 25 min strength training + 5 min of stretching + 30 min walk daily	NA	4	1
**Hoogeboom et al., 2010** [30]	Prehabilitation CON	Functional physical activities in the patient’s daily life + aerobic and strength ex. Usual care + advice	5 min walk + 25 min aerobic-ex + 25 min strength training	15-point perceived exertion scale	6	2
**McIsaac et al., 2022** [31]	Prehabilitation CON	Strength training + aerobic ex + flexibility + healthy food guide. Usual care + advice	1 h sessions: strength training + aerobic-ex + flexibility	NA	4	3
**Oosting et al., 2012** [26]	Prehabilitation CON	Functional activities and walking capacity Usual care + advice	Home ex with patient-adapted functional activities and walks	55–75% peak work rate	5	4

EX: exercise; min: minutes; and NA = not applicable.

**Table 3:** Grading of Recommendations Assessment, Development, and Evaluation (GRADE) for the 6 min walk test (6-MWT).

Certainty Assessment		N° of Participants		Summary of Findings		
№ of RCTs	Comparison	Intervention	Control	Effect–Relative (95% CI)	Certainty	* Reason
3	6-MWT (T1)	80/161	81/161	MD 9.71 (−38.92;58.36)	⬤⬤⬤◯ Moderate	Imprecision ^a^
2	6-MWT (T2)	70/140	154/584	MD −3.27 (−71.21;64.65)	⬤⬤◯◯ Low	Imprecision ^a^; Inconsistency ^b^
3	6-MWT (T3)	164/322	158/322	ES 15.01 (−22.05;52.073)	⬤⬤⬤◯ Moderate	Imprecision ^a^

Note: **95% CI:** 95% confidence interval; RCT: randomized controlled trial. * **Reason** refers to the GRADE system criteria that justify downgrading the quality of evidence for a specific outcome. ^a^ Imprecision: wide confidence intervals; ^b^ Inconsistency: heterogeneous results across studies.

**Table 4:** Grading of Recommendations Assessment, Development, and Evaluation (GRADE) for length of hospital stay (LOS).

Certainty Assessment		N° of Participants		Summary of Findings		
№ of RCT	Comparison	Intervention	Control	Effect–Relative (95% CI)	Certainty	* Reason
4	LOS	192/379	187/379	MD −0.46 (−0.96;0.03)	⬤⬤⬤◯ Moderate	Imprecision ^a^

Note: **95% CI:** 95% confidence interval; RCT: randomized controlled trial. * **Reason** refers to the GRADE system criteria that justify downgrading the quality of evidence for a specific outcome. ^a^ Imprecision: Wide confidence intervals.

## Data Availability

All the data used in this study were derived from publicly available institutional documents, news reports, and official statements. A list of sources and document references is available upon reasonable request from the corresponding author. No new datasets were generated or proprietary data were used during this study.

## Abbreviations

The following abbreviations are used in this manuscript:

ES	Effect size
LOS	Length of hospital stay
6-MWT	6 min walk test
SUCRA	Surface under the cumulative ranking

## Appendix A

### Appendix A.1

**Table A1:** Reporting elements for systematic reviews that incorporate network meta-analysis (PRISMA-N).

Section/Topic	Item	Checklist Item	Reported on Page
**TITLE**			
Title	1	Identify the report as a systematic review incorporating a network meta-analysis (or related form of meta-analysis).	*1*
**ABSTRACT**			
Structured summary	2	Provide a structured summary including the following, as applicable: **Background:** Main objectives. **Methods:** Data sources; study eligibility criteria, participants, and interventions; study appraisal; and synthesis methods, such as network meta-analysis. **Results:** Number of studies and participants identified; summary estimates with corresponding confidence/credible intervals; and treatment rankings may also be discussed. Authors may choose to summarize pairwise comparisons against a chosen treatment included in their analyses for brevity. **Discussion/Conclusions:** Limitations; conclusions; and implications of findings. **Other:** Primary source of funding; systematic review registration number with registry name.	*2*
**INTRODUCTION**			
Rationale	3	Describe the rationale for the review in the context of what is already known, including mention of why a network meta-analysis has been conducted.	*3*
Objectives	4	Provide an explicit statement of questions being addressed, with reference to participants, interventions, comparisons, outcomes, and study design (PICOS).	*3*
**METHODS**			
Protocol and registration	5	Indicate whether a review protocol exists and if and where it can be accessed (e.g., Web address); and, if available, provide registration information, including registration number.	*4*
Eligibility criteria	6	Specify study characteristics (e.g., PICOS, length of follow-up) and report characteristics (e.g., years considered, language, and publication status) used as criteria for eligibility, giving rationale. Clearly describe eligible treatments included in the treatment network and note whether any have been clustered or merged into the same node (with justification).	*4*
Information sources	7	Describe all information sources (e.g., databases with dates of coverage, contact with study authors to identify additional studies) in the search and date last searched.	*4*
Search	8	Present full electronic search strategy for at least one database, including any limits used, such that it could be repeated.	*4 and Table A2*
Study selection	9	State the process for selecting studies (i.e., screening, eligibility, included in systematic review, and, if applicable, included in the meta-analysis).	*4*
Data collection process	10	Describe method of data extraction from reports (e.g., piloted forms, independently, and in duplicate) and any processes for obtaining and confirming data from investigators.	*4, 5*
Data items	11	List and define all variables for which data were sought (e.g., PICOS, funding sources) and any assumptions and simplifications made.	4
**Geometry of the network**	**S1**	Describe methods used to explore the geometry of the treatment network under study and potential biases related to it. This should include how the evidence base has been graphically summarized for presentation and what characteristics were compiled and used to describe the evidence base to readers.	5,6
Risk of bias within individual studies	12	Describe methods used for assessing risk of bias of individual studies (including specification of whether this was performed at the study or outcome level) and how this information is to be used in any data synthesis.	5
Summary measures	13	State the principal summary measures (e.g., risk ratio, difference in means). Also describe the use of additional summary measures assessed, such as treatment rankings and surface under the cumulative ranking curve (SUCRA) values, as well as modified approaches used to present summary findings from meta-analyses.	6, 7
Planned methods of analysis	14	Describe the methods of handling data and combining results of studies for each network meta-analysis. This should include, but not be limited to: Handling of multi-arm trials; Selection of variance structure; Selection of prior distributions in Bayesian analyses; Assessment of model fit.	7
**Assessment of inconsistency**	**S2**	Describe the statistical methods used to evaluate the agreement of direct and indirect evidence in the treatment network(s) studied. Describe efforts taken to address its presence when found.	6, 7
Risk of bias across studies	15	Specify any assessment of risk of bias that may affect the cumulative evidence (e.g., publication bias, selective reporting within studies).	7
Additional analyses	16	Describe methods of additional analyses if performed, indicating which were prespecified. This may include, but not be limited to, the following: Sensitivity or subgroup analyses; Meta-regression analyses; Alternative formulations of the treatment network; Use of alternative prior distributions for Bayesian analyses (if applicable).	6, 7
**RESULTS**			
Study selection	17	Give numbers of studies screened, assessed for eligibility, and included in the review, with reasons for exclusions at each stage, ideally with a flow diagram.	and Figure
**Presentation of network structure**	**S3**	Provide a network graph of the included studies to enable visualization of the geometry of the treatment network.	Figures
**Summary of network geometry**	**S4**	Provide a brief overview of characteristics of the treatment network. This may include commentary on the abundance of trials and randomized patients for the different interventions and pairwise comparisons in the network, gaps of evidence in the treatment network, and potential biases reflected by the network structure.	7
Study characteristics	18	For each study, present characteristics for which data were extracted (e.g., study size, PICOS, and follow-up period) and provide the citations.	Table 1 and Appendix Tables
Risk of bias within studies	19	Present data on risk of bias for each study and, if available, any outcome level assessment.	7
Results of individual studies	20	For all outcomes considered (benefits or harms), present for each study (1) simple summary data for each intervention group and (2) effect estimates and confidence intervals. Modified approaches may be needed to deal with information from larger networks.	and figure and Appendex Material
Synthesis of results	21	Present results of each meta-analysis performed, including confidence/credible intervals. In larger networks, authors may focus on comparisons versus a particular comparator (e.g., placebo or standard care), with full findings presented in an appendix. League tables and forest plots may be considered to summarize pairwise comparisons. If additional summary measures were explored (such as treatment rankings), these should also be presented.	Appendix tables
**Exploration for inconsistency**	**S5**	Describe results from investigations of inconsistency. This may include such information as measures of model fit to compare consistency and inconsistency models, *P* values from statistical tests, or summary of inconsistency estimates from different parts of the treatment network.	Appendix Table
Risk of bias across studies	22	Present results of any assessment of risk of bias across studies for the evidence base being studied.	7
Results of additional analyses	23	Give results of additional analyses, if performed (e.g., sensitivity or subgroup analyses, meta-regression analyses, alternative network geometries studied, alternative choice of prior distributions for Bayesian analyses, and so forth).	9
**DISCUSSION**			
Summary of evidence	24	Summarize the main findings, including the strength of evidence for each main outcome; consider their relevance to key groups (e.g., healthcare providers, users, and policymakers).	9
Limitations	25	Discuss limitations at study and outcome level (e.g., risk of bias) and at review level (e.g., incomplete retrieval of identified research, reporting bias). Comment on the validity of the assumptions, such as transitivity and consistency. Comment on any concerns regarding network geometry (e.g., avoidance of certain comparisons).	10
Conclusions	26	Provide a general interpretation of the results in the context of other evidence and implications for future research.	11
**FUNDING**			
Funding	27	Describe sources of funding for the systematic review and other support (e.g., supply of data); the role of funders for the systematic review. This should also include information regarding whether funding has been received from manufacturers of treatments in the network and/or whether some of the authors are content experts with professional conflicts of interest that could affect use of treatments in the network.	No funding

### Appendix A.2

**Table A2:** Full search strategy for all the databases.

Pubmed	WOS	Cochrane Central
(“home-based” OR “preoperative prehabilitation” OR “preoperative exercise” OR “preoperative therapeutic”) AND (“comorbidities” OR “postoperative complications” OR mortality) AND (trial)	TS=((“home-based” OR “preoperative prehabilitation” OR “preoperative exercise” OR “preoperative therapeutic”) AND (“comorbidities” OR “postoperative complications” OR mortality) AND (trial))	(“home-based” OR “preoperative prehabilitation” OR “preoperative exercise” OR “preoperative therapeutic”) AND (“comorbidities” OR “postoperative complications” OR mortality) AND (trial)

### Appendix A.3

**Table A3:** The inclusion criterion was the use of these scales to assess frailty.

Groningen Frailty Indicator (GFI)	Clinical Frailty Scale (CFS)	Fried Frailty Index (FFI)	Frailty Index Identification of Seniors at Risk (ISAR)
The GFI is validated, with 15 items divided by 4 main domains (physical, cognitive, social, and psychological); the GFI scores between 0 and 15. People with scores of 4 and above are considered frail.	The CFS was introduced in the second clinical examination of the Canadian Study of Health and Aging (CSHA) as a way to summarize the overall level of fitness or frailty of an older adult after they had been evaluated by an experienced clinician.	The Fried frailty phenotype (FP) assesses physical frailty through five criteria: unintentional weight loss; weakness or poor handgrip strength; self-reported exhaustion; slow walking speed; and low physical activity.	It consists of six items that assess the presence or absence of risk factors for adverse outcomes after an emergency department visit. This tool predicts recurrent emergency visits, hospital admissions, functional decline, and long-term care facility admission at 30 and 180 days after the visit, and it correlates with functional dependence and depression.

### Appendix A.4

**Table A4:** Grading of Recommendations Assessment, Development, and Evaluation (GRADE) for the 6 min walk test (6-MWT).

Certainty Assessment						n of Participants		Effect	
№ of Studies	Study Design	Risk of Bias	Inconsistency	Indirectness	Imprecision	Intervention	Control	Relative (95% CI)	Importance
**6-MWT (T1)**									
3	randomized trials	not serious	not serious	not serious	serious	80/161	81/161	MD 9.71 (−38.92;58.36)	IMPORTANT
**6-MWT (T2)**									
2	randomized trials	not serious	serious ^a^	not serious	serious ^b^	70/140	70/140	MD -3.27 (−71.21;64.65)	CRITICAL
**6-MWT (T3)**									
3	randomized trials	not serious	not serious	not serious	serious	164/322	158/322	MD 15.01 (−22.05;52.073)	IMPORTANT

**CI:** confidence interval; ^a^ inconsistency is serious because the results show wide variability; and ^b^ wide confidence interval.

### Appendix A.5

**Table A5:** Grading of Recommendations Assessment, Development, and Evaluation (GRADE) for length of hospital stay (LOS).

Certainty Assessment						n of Participants		Effect	
**№ of Studies**	Study Design	Risk of bias	Inconsistency	Indirectness	Imprecision	Intervention	Control	Relative (95% CI)	Importance
4	randomized trials	not serious	not serious	not serious	serious	192/379	187/379	MD −0.46 (−0.96;0.03)	IMPORTANT

### Appendix A.6

**Table A6:** Sensitivity analysis by comparison groups for *the 6MWT*.

*T1–T2*			
**Reference**	**ES**	**LL**	**UL**
**Carli et al., 2020** [29]	11.130	−72.600	94.860
**Hoogeboom et al., 2010** [30]	11.103	−40.199	62.407
**Oosting et al., 2012** [26]	7.464	−48.200	63.129
***T1–T3***			
**Reference**	**ES**	**LL**	**UL**
**Carli et al., 2020** [29]	5.730	−42.830	54.290
**McIsaac et al., 2022** [31]	7.116	−50.618	64.852
**Oosting et al., 2012** [26]	25.398	−15.554	66.351

### Appendix A.7

**Table A7:** Sensitivity analysis by comparison groups for *length of hospital stay* (LOS).

Reference	ES	LL	UL
**Berkel et al., 2022** [28]	−0.460	−0.959	0.039
**Carli et al., 2020** [29]	−0.314	−1.445	0.815
**McIsaac et al., 2022** [31]	−0.470	−0.969	0.028
**Oosting et al., 2012** [26]	−0.496	−1.037	0.044
